# Osteosynthesis of bilateral Vancouver B2 periprosthetic femoral fracture after a bilateral RM^®^ total hip arthroplasty at 24 and 21-years follow-up: A case report

**DOI:** 10.1016/j.ijscr.2019.05.009

**Published:** 2019-05-11

**Authors:** Isabel Dinis Ferreira, João Cura Mariano, Francisco M. Lucas, Fernando M. Judas

**Affiliations:** Orthopedics Department, Centro Hospitalar e Universitário de Coimbra (CHUC), Faculty of Medicine, University of Coimbra, Praceta Prof. Mota Pinto, 3000-075, Coimbra, Portugal

**Keywords:** Bilateral periprosthetic fractures, Isoelastic total hip arthroplasty, Vancouver B2, Locking compression plate, Femoral stem loosening

## Abstract

**Introduction:**

The management of periprosthetic femoral fractures following hip arthroplasty is challenging, and the choice between osteosynthesis of the fracture and the revision of the prosthesis is still matter of discussion.

**Case report:**

In a 81-year-old male patient, a bilateral Vancouver type-B2 periprosthetic femoral fracture with stem loosening occurred after an accidental fall. The patient had severe medical comorbidities. The radiographic study showed a bilateral Robert Mathys cementless total hip arthroplasty at 24 and 21-years follow-up. The fractures were treated with open reduction and fixation with locking compression plates. Bicortical fixation of the loose stem was obtained by the screws of the locking plate, due to the polymeric composition of the isoelastic femoral stem. Both fractures sites were augmented with bone allografts. At follow-up period of 12 months, the X-rays showed bone union of both fractures and bilateral stable stem fixation. The patient expressed high degree of satisfaction with surgery result.

**Discussion:**

The standard treatment for Vancouver type-B2 periprosthetic femoral fractures is the removal of the loose implant, fixation of the fracture, and implantation of a new revision femoral stem. However, the implantation of two long revision hip prostheses is a major operation for an older patient with precarious health condition, which can contribute to higher risk of medical and prosthetic complications.

**Conclusion:**

In older patients with multiple comorbidities, the use of locking plates can be a valid treatment of bilateral Vancouver B2-periprosthetic femoral fractures following RM^®^ cementless isoelastic stem, as an alternative surgical option to femoral stem revision.

## Highlights

• The management of periprosthetic femoral fractures following hip arthroplasty is challenging.
• Vancouver type-B2 periprosthetic femoral fractures require revision arthroplasty by replacement of the femoral component.
• In older patients with multiple comorbidities the osteosynthesis of Vancouver type-B2 periprosthetic femoral fracture is a valid treatment.
• Anatomically fracture reduction, the use of locking compression plates, and the preservation of the hip joint, are the key-points to a successful outcome.

## 1 Introduction

The incidence of periprosthetic femoral fractures is increasing, likely due to the larger number of total knee and hip arthroplasties being performed and the increased survivorship of the arthroplasty population. Patients with periprosthetic fractures are typically elderly and frail and have osteoporosis [1,2].

The choice of treatment modalities depends on various primary factors: the condition of the patient, the location of the fracture, the stability of the implant, and the quality of underlying bone stock, among others. No clear consensus exists regarding the optimal management strategy because there is limited high-quality research [3].

Nonoperative management is reserved for cases of stable trochanteric fractures around a well-fixed implant (Vancouver type-A), or those in patients who are unable to undergo surgical treatment, and can lead to problems related to decreased functional status and longer rehabilitation. Operative management has the advantages of early mobilization and reduced hospital stay. It also provides a reduction in systemic and local complications such as malunion and nonunion [4].

A comprehensive approach to these challenging patients, including identification of surgical risks, proper surgical planning, and rehabilitation may lead to improved outcomes. The Vancouver classification facilitates treatment decisions [5].

In the presence of a stable prosthesis (types B1 and C fractures), most authors recommend surgical stabilization of the fracture with plates, strut grafts, or a combination of them. For fractures around a loose femoral prosthesis (types B2 and B3), revision using an extensively porous-coated uncemented long stem, with or without additional fracture fixation, appears to offer the most reliable outcome [6].

The purpose of this paper was to show the outcome of bilateral Vancouver type-B2 periprosthetic femoral fracture treated with open reduction and internal fixation, using a locking compression plate and bone allografts, in an older patient with multiple comorbilities.

This work has been reported in line with the SCARE criteria [7].

## 2 Case report

**Fig. 1:**
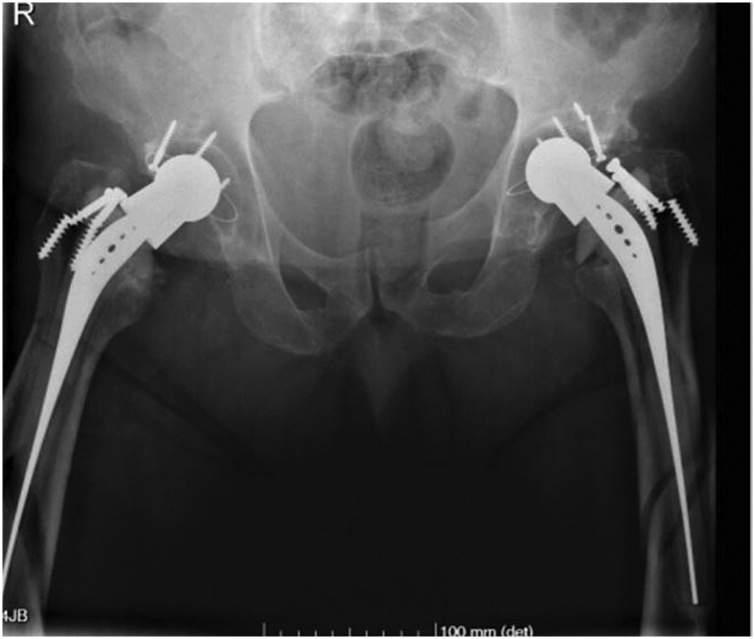
Preoperative anteroposterior radiograph of the pelvis showing bilateral Vancouver type-B2 periprosthetic femoral fracture.

A 81-year-old male was victim of an accidental fall from a height of 5 m. The patient referred bilateral groin and thigh pain, clinical examination showed inability to actively move the legs and pain on passive movement. The radiographic studies showed a bilateral fracture of the femoral shaft, and a bilateral Robert Mathys (RM^®^) cementless total hip arthroplasty. The bone lesions were classified as Vancouver type-B2 periprosthetic femoral fracture (Fig. 1).

**Fig. 2:**
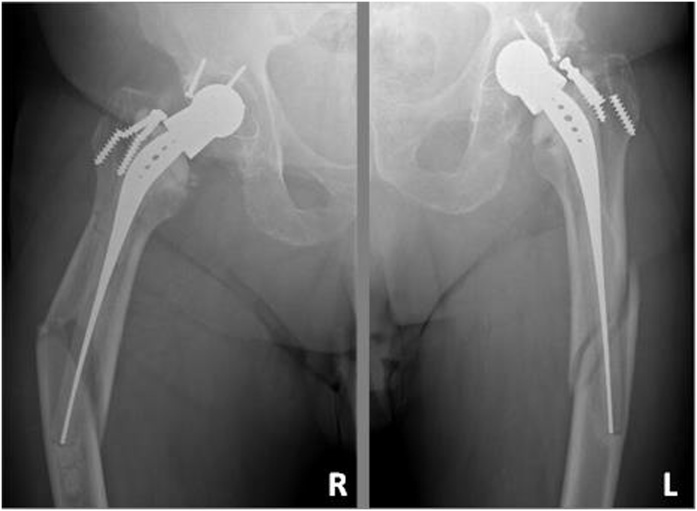
Preoperative anteroposterior radiographs of the hips showing bilateral isoelastic prosthesis with osteolysis along the whole length of the femoral stem after 24 and 21 years in situ. Broken screws due to the prosthetic instability.

The patient had a body mass index (BMI) of 33 kg/m^2^ and a notable comorbilities. On the right hip, a RM^®^ cementless total hip arthroplasty (isoelastic polyacetal stem with stainless-steel head and polyethylene cementless acetabular cup) was implanted, with 24-years follow-up. On the left side, the anteroposterior radiograph showed also a RM^®^ cementless total hip arthroplasty, with 21-years follow-up. On both sides it was possible to observe a biological process of acetabular polyethylene wear, instability of the femoral stem with broken femoral screws, and Paprosky type II femoral osteolysis (Fig. 2).

**Fig. 3:**
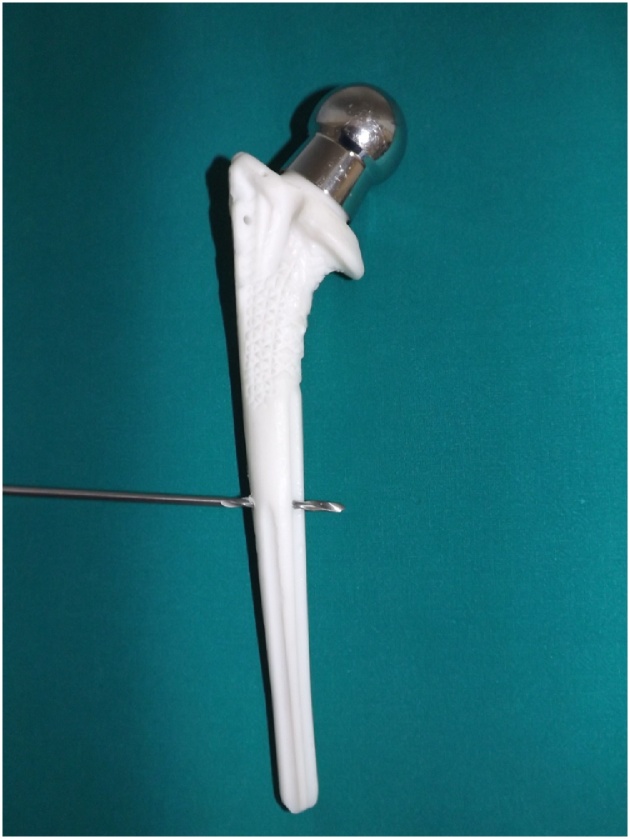
The femoral component of the third-generation isoelastic RM^®^ total hip replacement is easily perforated with a 3.2 mm drill due to their polymeric composition.

The fractures were treated with open reduction and fixation with a distal femur locking compression plate (LCP^®^), with a combination of 3.5 mm nonlocking and locking screws. Therefore, a right distal LCP^®^ plate was applied on left side and a left distal LCP® plate was used on the right side. The femoral stem was easily perforated with a 3.2 mm drill because the implant is composed by a polymer (Fig. 3), a polyacetal resin, with a stainless steel core to avoid over-elasticity in the neck region [8]. The fractures sites were augmented with criopreserved morselized cancellous bone allografts from the Bone and Tissue Bank of our institute [9]. On the right side a criopreserved structural fibular bone allograft was also applied on the anterior surface of the femur and was fixed with two cerclage wires.

The surgery was performed in the lateral decubitus, in two phases. First the right femur was operated, then the patient was repositioned to operate the left side. The surgery was performed without the use of a pneumatic tourniquet. The time of the surgery was 2 h and 15 min. Using a Cell-Saver System^®^ the total blood loss was 250 cc. No intraoperative difficulties in patient management were found.

No complications were reported in the perioperative course or during the hospitalization period. The postoperative course showed no problems with respect to the hips. The patient was submitted to an intensive rehabilitation protocol included early mobilization and walking with two crutches.

At 4-months follow-up, the patient presented stable hips and the radiographs showed signs of bone union of the fractures. He reported moderate pain, and some limitation of ordinary activity.

**Fig. 4:**
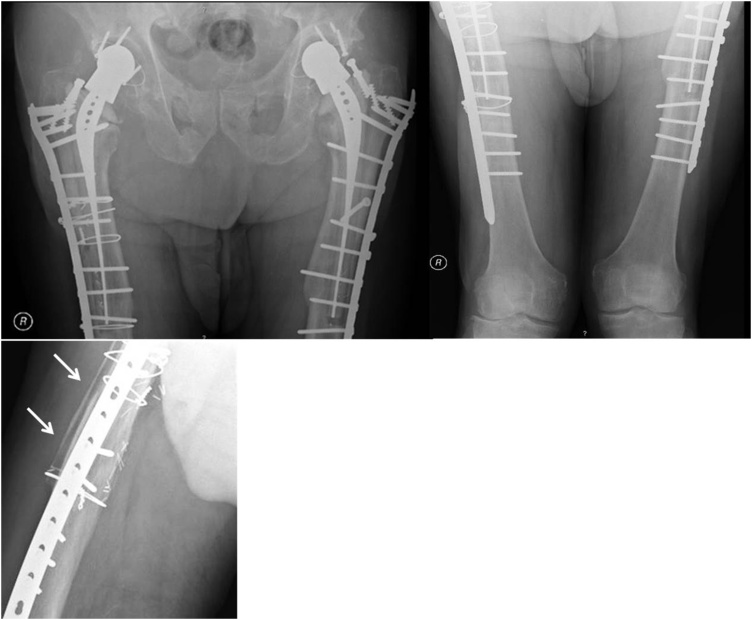
Postoperative radiograph images of the pelvis and of bilateral femur after open reduction and ORIF with locking compression plates. At follow-up period of 12 months, the X-rays showed bone union of both fractures and bilateral stable stem fixation. The lateral radiograph of the right femur showed also no signs of bone resorption of the fibular allograft.

At 12-months follow-up, the patient presented an asymptomatic hips and expressed high degree of satisfaction with surgery result. He reported no pain in his thighs. The femoral radiograph showed consolidation of both fractures and the fibular structural allograft had no signs of bone resorption (Fig. 4). The patient was clinically able to walk without external support.

## 3 Discussion

Periprosthetic femoral fractures following hip arthroplasty are most frequently caused by minor trauma such as a fall, but does occur, although rarely, in the absence of trauma, implying that this condition may be associated with risk factors including old age, comorbidities, osteoporosis, loosening of prosthesis, and osteolysis [1,10].

These fractures are associated with a high rate of complication and a poor union rate if treated in nonoperative fashion. Their management is challenging and the choice between osteosynthesis of the fracture and the revision of the prosthesis is still matter of discussion [4,6]. The prevention of periprosthetic femoral fractures remains the best strategy.

Simultaneous periprosthetic femoral fracture is a rare complication. In this report the fractures were caused by a high energy fall in a patient with predisposing factors: old age, excessive weight body and loosening of both femoral prostheses. The fractures were classified as type-B2 according to the Vancouver classification, the implant is loose but has sufficient bone stock for straightforward revision surgery [5].

The established scientific principles recommends that Vancouver type-B2 periprosthetic femoral fractures require revision arthroplasty by replacement of the femoral component with a non-cemented, long stem that bypasses the fracture line, because a higher rate of re-operation were associated when the femoral prosthesis is not replaced [3,10].

However, our patient presented advanced age and excessive body mass index associated with severe medical comorbidities: venous insufficiency of the lower legs with severe skin trophic changes and sequelae of thrombophlebitis, vascular deficits of the lower limbs, atherosclerosis, femoral prosthesis by aortic aneurysm, atrial fibrillation, chronic obstructive pulmonary disease, metabolic syndrome, recurrent abdominal hernias, prostatic hyperplasia, among others.

Although current surgical treatment algorithms, commonly recommend open reduction and internal fixation (ORIF) solely for fractures with a stable femoral stem [11,12], in our patient we considered the osteosynthesis of the fractures as the most recommended treatment. The implantation of two long revision hip prostheses is a prolonged and major operation for an older patient with precarious health condition, which can contribute to higher risk of medical and prosthetic complications, and difficulties at functional rehabilitation.

Modern internal fixation is frequently achieved with locking plates, which provide relative fracture stability, and potentially preserve the periosteal blood supply to the fractured bone, especially when minimally invasive surgery and indirect fracture reduction techniques are used [13]. Patients treated with ORIF had a significant shorter skin-to-skin surgical time and fewer perioperative blood transfusions. There were more complications reported in the revision arthroplasty cohort compared to patients that were treated with ORIF [14].

In the other hand, open reduction and internal fixation utilizing locking compression plates (LCP^®^) might be an effective treatment with a reduced surgical time and less complex procedure in a typically elderly patient with multiple comorbidities [15].

Rigid fixation for periprosthetic femoral fractures with screws and plates is challenging due to interference of a preexisting femoral stem. In our case, it was possible to perforate the implant due to the polymeric composition of the isoelastic femoral stem (synthetic resin), and an excellent bicortical fixation of the loose stem was obtained by the screws of the locking plate. The distal part of the plates were fixed with at least eight cortices as recommended by other authors [16].

Despite being not a mandatory indication in this case, morselized cancellous bone allografts were used in both fractures and a fibular bone allograft was applied on the right side. Our institute has a Bone and Tissue Bank [9], with thirty-seven years of clinical experience. Bone grafts associated with metallic plates reduces stress shielding, increases the probability of fracture consolidation, makes the system more stable, reduces complications, and improves patient quality of life due to a shorter functional recovery, compared to internal fixation with a simple plate. Cortical strut grafts offer reliable fracture healing [17].

RM^®^ isoelastic total hip replacement was one of the earliest cementless designs (Morscher and Mathys 1974, 1975). The concept of isoelasticity was based on the assumption that the implant and the bone should deform as one unit to avoid stress shielding. However, the performance of this prosthesis was unacceptably poor. Higher debris production and poor primary fixation are believed to be the main reason for the high failure rate [8]. Therefore, the femoral implant was abandoned.

Despite isoelastic prosthesis has been discontinued, we believe that many femoral stems will continue in situ, similarly to the present report. So the importance of this paper is to show the good clinical and radiological outcomes of the conservative surgical approach used for the treatment of a bilateral Vancouver type-B2 periprosthetic femoral fracture, after a bilateral total hip arthroplasty with 24 and 21 years-follow-up.

A bicortical stable fixation of the loose femoral stems by the screws of the locking plates were possible, due to the polymeric composition of the stem. At 12-months follow-up, the patient referred absence of pain on full weight-bearing and was clinically able to walk without external support, and expressed high degree of satisfaction with surgery result.

To our knowledge, there are no previous reports concerning the possibility of the restoration of the stem mechanical stability by the plate screws, in this type of femoral prosthesis, avoiding the implantation of a new revision femoral prosthesis.

## 4 Conclusion

In older patients with multiple comorbidities, the use of locking plates is a valid treatment of bilateral Vancouver B2 periprosthetic femoral fractures following RM^®^ cementless isoelastic stem, as an alternative option to femoral stem revision. The key-points to a successful outcome are anatomical fracture reduction, stable fixation of the stem by the screws of the plate avoiding stem subsidence, and the preservation of the hip joint avoiding the risk of prosthesis dislocation.

## Conflicts of interest

All authors declare that there are not any competing interests.

## Funding

There is no source of funding.

## Ethical approval

This study is exempt from ethical approval in the authors’ institution. The authors have followed the protocols of their work center on the publication of data.

## Consent

Written informed consent was obtained from the patient for publication of this case report and any accompanying images. A copy of the written consent is available for review by the Editor-in-Chief of this journal on request.

## Author contribution

All authors have participated in the surgery and were responsible for the care of the patient.

All authors contributed to the collection of data and drafted the manuscript.

All authors reviewed and revised the manuscript for submission.

## Registration of Research Studies

Not applicable.

## Guarantor

Isabel Dinis Ferreira, Fernando Judas.

## Provenance and peer review

Not commissioned, externally peer-reviewed.

## References

[1] Lindahl H., Malchau H., Herberts P., Garellick G. (2005). Periprosthetic femoral fractures classification and demographics of 1049 periprosthetic femoral fractures from the Swedish National Hip Arthroplasty Register. J. Arthroplasty.

[2] Schmidt A.H., Kyle R.F. (2002). Periprosthetic fractures of the femur. Orthop. Clin. N. Am..

[3] Khan T., Grindlay D., Ollivere B.J., Scammell B.E., Manktelow A.R., Pearson R.G. (2017). A systematic review of Vancouver B2 and B3 periprosthetic femoral fractures. Bone Jt. J..

[4] Rayan F., Haddad F. (2010). Periprosthetic femoral fractures in total hip arthroplasty—a review. Hip Int..

[5] Duncan C.P., Masri B.A. (1995). Fractures of the femur after hip replacement. Instr. Course Lect..

[6] Marsland D., Mears S.C. (2012). A review of periprosthetic femoral fractures associated with total hip arthroplasty. Geriatr. Orthop. Surg. Rehabil..

[7] Agha R.A., Borrelli M.R., Farwana R., Koshy K., Fowler A., Orgill D.P., For the SCARE Group (2018). The SCARE 2018 statement: updating consensus Surgical CAse REport (SCARE) guidelines. Int. J. Surg..

[8] Trebse R., Milosev I., Kovac S., Mikek M., Pisot V. (2005). Poor results from the isoelastic total hip replacement: 14–17-year follow-up of 149 cementless prostheses. Acta Orthop..

[9] Judas F., Teixeira L., Proença A. (2005). Coimbra University Hospitals’ bone and tissue bank: twenty-two years of experience. Transplant. Proc..

[10] Dargan D., Jenkinson M.J., Acton J.D. (2014). A retrospective review of the Dall-Miles plate for periprosthetic femoral fractures: twenty-seven cases and a review of the literature. Injury.

[11] Duwelius P.J., Schmidt A.H., Kyle R.F., Talbott V., Ellis T.J., Butler J.B. (2004). A prospective, modernized treatment protocol for periprosthetic femur fractures. Orthop. Clin. N. Am..

[12] Kim M.W., Chung Y.Y., Lee J.H., Park J.H. (2015). Outcomes of surgical treatment of periprosthetic femoral fractures in cementless. Hip Arthroplasty Hip Pelvis.

[13] Ricci W.M., Bolhofner B.R., Loftus T., Cox C., Mitchell S., Borrelli J.Jr. (2005). Indirect reduction and plate fixation, without grafting, for periprosthetic femoral shaft fractures about a stable intramedullary implant. J. Bone Jt. Surg. Am..

[14] Solomon L.B. (2015). Is internal fixation alone advantageous in selected B2 periprosthetic fractures?. ANZ J. Surg..

[15] Joestl J., Hofbauer M., Lang N., Tiefenboeck T., Hajdu S. (2016). Locking compression plate versus revision-prosthesis for Vancouver type B2 periprosthetic femoral fractures after total hip arthroplasty. Injury.

[16] Buttaro M.A. (2007). Locking compression plate fixation of Vancouver type-B1 periprosthetic femoral fractures. J. Bone Jt. Surg. Am..

[17] Chandler H.P., King D., Limbird R. (1993). The use of cortical allograft struts for fixation of fractures associated with well-fixed total joint prostheses. Semin. Arthroplasty.

